# Meta-analysis of the effect of vitamin D on depression

**DOI:** 10.3389/fpsyt.2025.1622796

**Published:** 2025-07-31

**Authors:** Lu Wang, Siyuan Su, Yongsheng Liu

**Affiliations:** ^1^ School of Medical Imaging, Qilu Medical University, Jinan, Shandong, China; ^2^ School of Clinical Medicine, Qilu Medical University, Jinan, Shandong, China

**Keywords:** vitamin D, depression, therapy, meta-analysis, randomized controlled trials, symptom relief

## Abstract

**Objective:**

To investigate the effect of vitamin D supplementation on depressive symptoms by conducting a meta-analysis of randomized controlled trials.

**Methods:**

We systematically searched PubMed, EMBASE, the Cochrane Library, CNKI, VIP, Wanfang, and additional databases for relevant literature published between January 1, 2000, and October 31, 2024. Two independent reviewers screened titles, abstracts, and full texts, extracted data, and assessed study quality. RevMan 5.3 software was employed to calculate pooled effect sizes and assess heterogeneity.

**Results:**

Twenty trials met our inclusion criteria. Meta-analysis using a random-effects model demonstrated that vitamin D supplementation significantly reduced depressive symptom scores compared to controls (standardized mean difference [SMD] = –0.36; 95% confidence interval [CI], –0.52 to –0.20; P < 0.00001).

**Conclusion:**

Our findings indicate that vitamin D supplementation is associated with a moderate but statistically significant improvement in depressive symptoms. These results support the potential role of vitamin D as an adjunctive treatment for depression, particularly in individuals with baseline deficiency.

## 1 Introduction

Depression is a common psychological disorder that significantly impacts both the physical and mental well-being of individuals. According to the World Health Organization, over 300 million people worldwide are affected by depression (1). It not only causes great suffering to patients but also places a heavy burden on families and society. Common treatments for depression include medication, psychotherapy, and somatic treatments. In terms of drug treatment, selective serotonin reuptake inhibitors (SSRIs), serotonin, and norepinephrine reuptake inhibitors (SNRIs) (2) can alleviate symptoms, but some patients have adverse reactions and poor efficacy. Psychotherapies, such as cognitive behavioral therapy, can help patients change negative thought patterns (3), but their effectiveness in major depression alone is limited. Somatic treatments, like electroconvulsive therapy, is indicated in certain patients with severe disease, but it has some side effects (4). Therefore, the adjuvant treatment of patients with depression has become an urgent problem to be solved.

Vitamin D exists in two distinct forms, namely vitamin D_2_ and vitamin D_3_, and undergoes a process of hydroxylation twice within the body, facilitated by the liver and kidneys, to become biologically active. Upon exposure to ultraviolet light, 7-Dehydrocholesterol present in the skin undergoes a transformation into vitamin D_3_, while dietary sources also provide vitamin D. Vitamin D is conveyed through the bloodstream by a specific binding protein, ultimately arriving at the liver, where it undergoes further hydroxylation to form 1,25-dihydroxyvitamin D_3_, the biologically active variant of vitamin D (5). In previous studies, the potential link between vitamin D and depression has gradually attracted attention. Vitamin D not only plays a key role in calcium and phosphorus metabolism and bone health but is also involved in a variety of physiological processes, including the development and functional regulation of the nervous system (6).

There exists a notable prevalence of vitamin D deficiency among individuals experiencing depression, with diminished levels of this vitamin correlating with the intensity of depressive symptoms. However, clinical studies on the effect of vitamin D supplementation on depressive symptoms have been inconsistent. Some studies have shown that vitamin D supplementation improves symptoms of depression, while others have found no significant effect. Drawing firm conclusions is challenging due to differences in sample size, participant characteristics, vitamin D supplementation dosage, and duration of treatment.

The current treatment of depression has limitations, and vitamin D is of great significance as a potential intervention factor. Recent years have seen a proliferation of systematic reviews and meta-analyses examining the relationship between vitamin D and depression, yet their conclusions remain heterogeneous. An umbrella meta-analysis by Musazadeh et al. (2022) synthesized ten RCT-based meta-analyses and found a pooled SMD of –0.40 (95% CI –0.60 to –0.21) for depressive symptoms, alongside observational evidence linking low 25(OH)D to higher odds of depression (OR 1.60; 95% CI 1.08–2.36) (7). Xie et al. (2022) included 29 randomized controlled trials (RCTs) (n=4,504) and reported benefit both for prevention (SMD –0.23) and treatment (SMD –0.92), especially at doses >2,800 IU/day and durations ≥8 weeks. More recently (8), Ghaemi et al. (2024) conducted a dose–response meta-analysis of 31 trials (n=24,189), demonstrating that each additional 1,000 IU/day of vitamin D3 yielded an SMD of –0.32 (95% CI –0.43 to –0.22) and that short-term supplementation (≤8 weeks) produced stronger effects than longer regimens (9). Despite these insights, existing reviews have not uniformly explored how baseline 25(OH)D status, vitamin D form (D_2_ *vs*. D_3_), administration route, or dosing regimens moderate treatment effects. In contrast, our study integrates comprehensive subgroup analyses and meta-regression to identify which patient characteristics and supplementation strategies most strongly predict efficacy, thereby offering more precise, mechanism-guided recommendations for clinical practice. Through this meta-analysis, we sought to answer the following key question: Does vitamin D supplementation improve depressive symptoms in adults, and how are these effects moderated by factors such as baseline 25(OH)D status, supplementation dose, form (D_2_ *vs*. D_3_), route of administration, and treatment duration? By integrating existing randomized controlled trials, we aim not only to quantify the overall efficacy of vitamin D for depression but also to identify which patient characteristics and intervention strategies yield the greatest clinical benefit. These insights may suggest new adjunctive approaches to overcome current treatment limitations—such as efficacy plateaus and adverse effects—and will inform the design of future large-scale RCTs and mechanistic studies.

## 2 Methods

### 2.1 Search strategy and study selection

**Figure 1:**
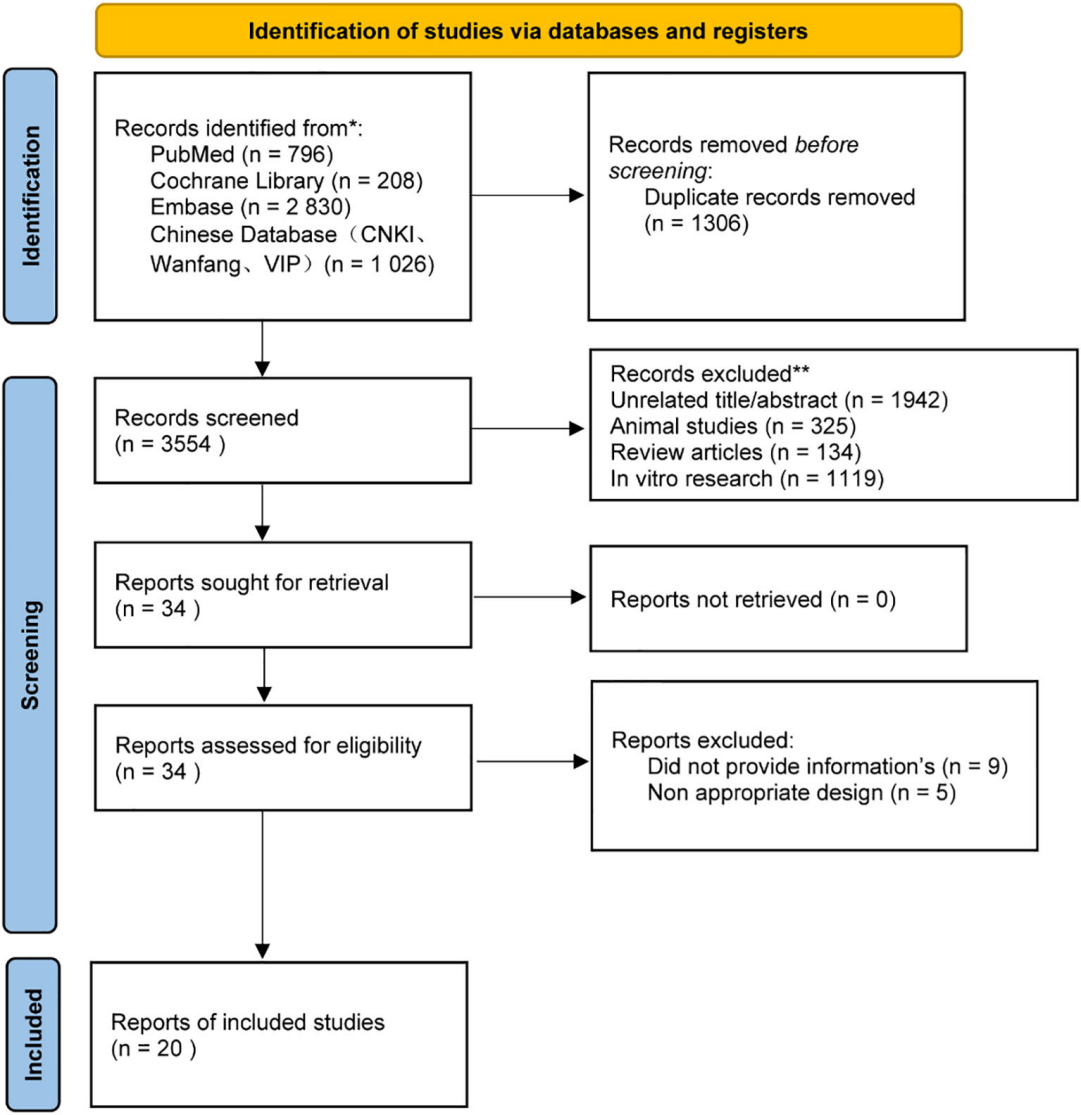
PRISMA Flow Diagram. PRISMA (Preferred Reporting Items for Systematic Reviews and Meta-Analyses) flow diagram for studies included in and excluded from the meta-meta-analysis.

We conducted a comprehensive literature search for randomized controlled trials of vitamin D supplementation in depression, covering January 1, 2000, through October 31, 2024. The following databases were queried: PubMed, EMBASE, Cochrane Library, CNKI, VIP, Wanfang, and others. The search was executed by integrating subject headings with free words, tailored to the distinct characteristics of each database. The screening and selection process is illustrated in the PRISMA flowchart shown in **Figure 1**.

The search terms in Chinese were: “vitamin D,” “calcitriol,” “25-hydroxyvitamin D,” “depression,” etc. The English search terms were: “vitamin D,” “25(OH)D,” “depressive symptoms,” “emotional depression,” “depressive episode,” “depressive disorder,” etc. (PROSPERO registration number: CRD42024597870). Full search strategies for each database, with exact syntax and hit counts, are provided in **Supplementary Table S1**. Two review authors independently screened titles/abstracts and full texts, extracted data, and assessed risk of bias using the Cochrane tool. Discrepancies were resolved by a third review author.

### 2.2 Inclusion and exclusion criteria

Inclusion criteria were as follows: (1) randomized controlled trials conducted between January 1, 2000, and October 31, 2024; (2) adult participants (≥18 years) diagnosed with major depression or exhibiting “depressive tendencies” (operationalized as baseline scores above scale-specific cutoffs, e.g., ≥8 on Hamilton Depression Rating Scale (HAMD), Beck Depression Inventory (BDI), or Patient Health Questionnaire-9 (PHQ-9), or equivalent thresholds on Geriatric Depression Scale (GDS), Edinburgh Postnatal Depression Scale (EPDS), or Depression, Anxiety and Stress Scale (DASS)), regardless of gender, ethnicity, or physical comorbidities; (3) interventions using native vitamin D (D_2_ or D_3_), administered orally or by injection, compared against placebo or treatment-as-usual; and (4) reporting pre- and post-intervention means and standard deviations for any validated depressive symptom scale. Detailed information on all included trials—including baseline 25(OH)D status, scale(s) used, and pre/post scores—is provided in **Supplementary Table S2**.

Exclusion criteria included: (1) non-RCT designs (animal or *in vitro* studies, case reports, observational cohorts) or trials without a control arm; (2) trials in which depression was not the primary indication (e.g., primary anxiety disorders or insomnia) or where participants’ principal psychiatric condition lay outside depression; and (3) use of vitamin D analogues rather than native vitamin D_2_ or D_3_ preparations.

### 2.3 Data extraction and quality assessment

The articles underwent independent screening by two authors, who meticulously extracted data and evaluated their quality through the application of the Cochrane Collaboration’s risk of bias tool. Eligible studies were extracted based on the following characteristics: year of publication, name of the first author, study design, study location, age, gender (self-reported), body composition or metabolic profile of study subjects, and relevant ANOVA measures. The tool relies on information in the following areas, including allocation concealment, blinding of participants and raters, completeness of outcome data, selective reporting of study results, and other sources of bias. There are three levels of risk: High Risk, Unclear Risk, and Low Risk. The third author resolved the disagreement between the two authors.

### 2.4 Data synthesis and statistical analysis

The mean and the standard deviation (SD) of the intervention and placebo groups were obtained from the studies included in the analysis. Data analysis was performed using RevMan 5.3. Weighted mean difference (WMD) was used as the effect measure for continuous outcome variables using the same measurement tool; conversely, standardized mean difference (SMD) was used as the effect indicator.

Ninety-five percent confidence intervals (CIs) were calculated for each effect size. Statistical heterogeneity was assessed using the Chi-square test and I² statistic. When I² was less than 50%, indicating low heterogeneity among the studies, a fixed-effect model was employed for the meta-analysis. Conversely, when the Chi-square test P-value was less than 0.05 or I² exceeded 50%, indicating substantial heterogeneity, a random-effects model was utilized.

We selected the HAMD, BDI, and PHQ-9 because they are the most commonly employed, validated measures of depressive symptom severity in RCTs and are recommended by clinical guidelines for evaluating treatment response. Continuous symptom scales (rather than categorical diagnoses such as SCID/DSM-based MDD) enable calculation of standardized effect sizes, and their frequent use across studies maximizes comparability. Other instruments (e.g., MMPI, PAI, PHQ-8) were less consistently reported and therefore addressed in sensitivity analyses rather than the primary analysis.

We conducted subgroup analyses considering a range of factors such as vitamin D dosage, intervention duration, the presence or absence of concurrent physical activity, sex ratio, the existence of medical conditions, and the size of the study sample to identify potential sources of heterogeneity.

We also performed a sensitivity analysis in which each study was excluded to examine its effect on pooled point estimates. The outcomes of at least 10 meta-analyses were scrutinized through formal assessments, focusing on the effects derived from small-scale studies. If these assessments indicated potential issues, a visual evaluation of the funnel plot was conducted. If asymmetries were observed in the funnel plot and publication bias was suspected, trimming and filling methods were applied to assess the impact of potentially missing small studies on the overall effect.

### 2.5 Publication bias

**Figure 2:**
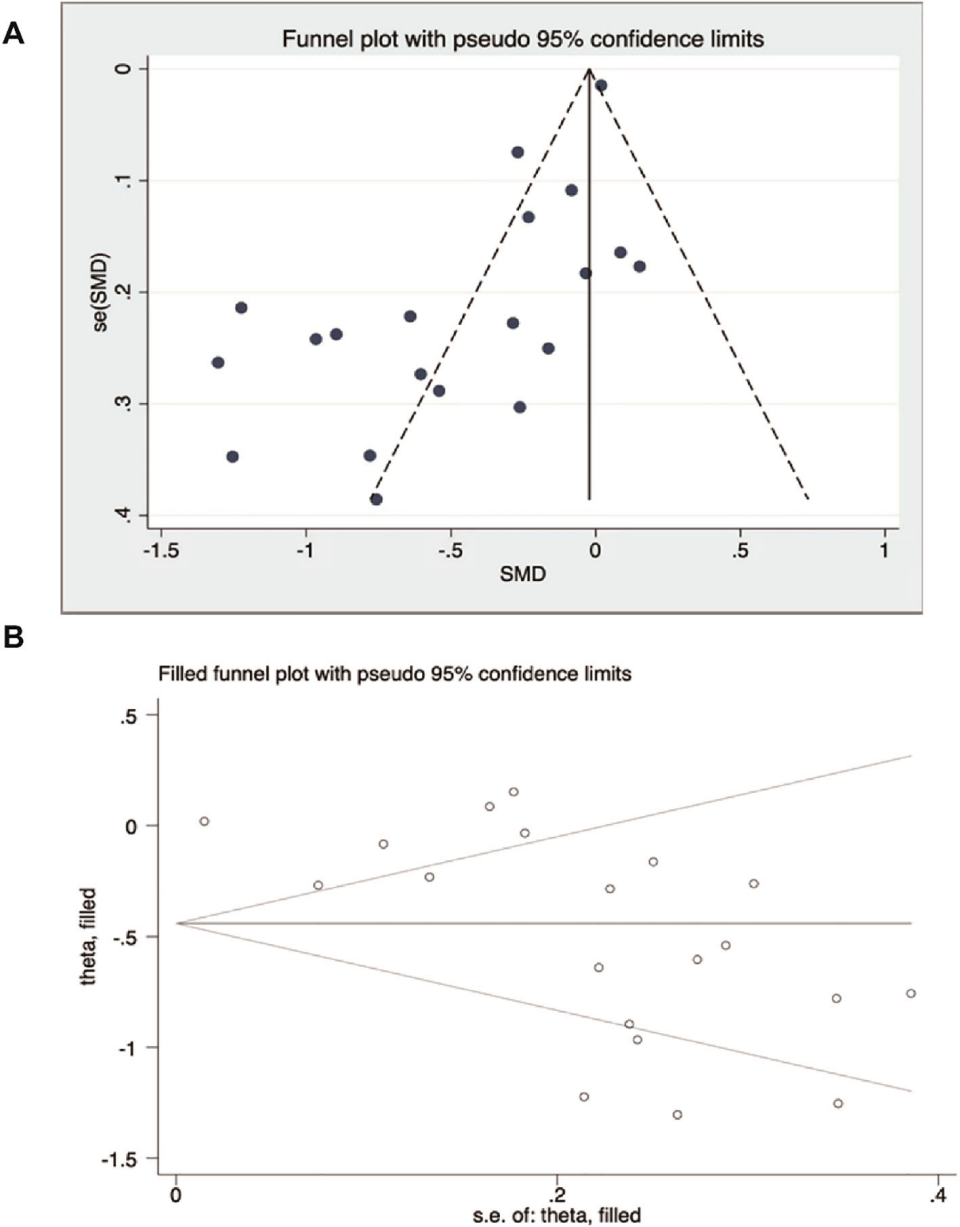
Publication bias analysis. **(A)** Funnel plot of observed studies. **(B)** Trim-and-fill funnel plot (no missing studies were imputed).

A total of 20 RCTs were included in this study, and the total number of scoring articles in this study was >10, so funnel plots were used to evaluate publication bias in the total studies. Visual inspection of the funnel plot indicated asymmetry (**Figure 2A**), suggesting potential bias. Egger’s regression test confirmed this (P<0.001, **Supplementary Table S3**). To address bias, the trim-and-fill method was applied and zero “missing” studies were identified. The effect size remained unchanged before and after the trim-and-fill method (**Figure 2B**).

## 3 Result

### 3.1 Preliminary search

A preliminary search identified 4860 articles, of which 1306 were duplicates. After eliminating redundant studies and those deemed irrelevant, 34 articles underwent full-text review for eligibility. Two independent reviewers assessed the full texts, and any discrepancies were resolved through discussion or by a third reviewer. Following this process, 20 studies met the inclusion criteria and were included in the final analysis. (10–29) All included studies were full-text publications. Articles were excluded for the following reasons: animal studies, *in vitro* studies, reviews, and non-randomized controlled trials (non-RCTs).

### 3.2 Data extraction and quality assessment

**Figure 3:**
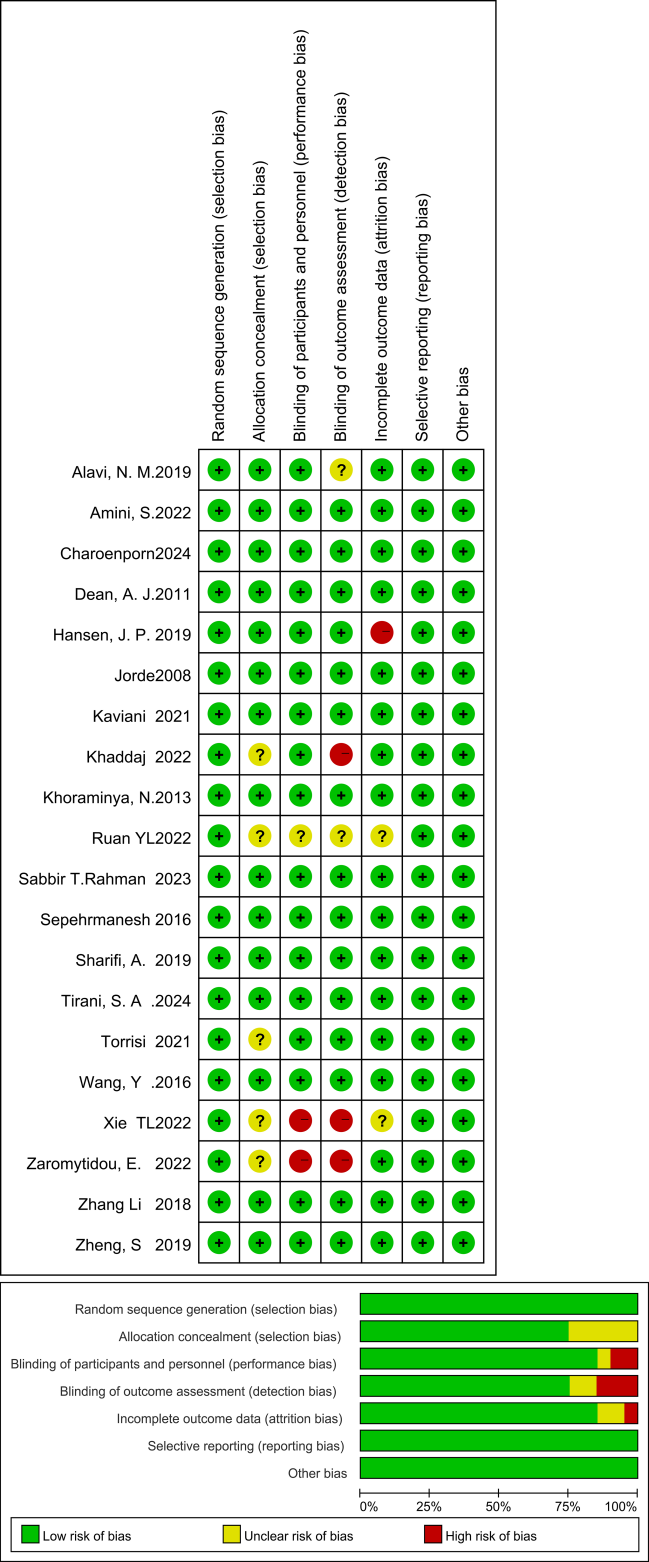
Risk-of-Bias summary.

These studies performed well in the generation of random sequences and the concealment of allocation. The majority of the studies had a low risk of bias, indicating that the methods of randomization and allocation concealment were relatively standardized. However, there was a relatively high proportion of high risk or unclear risk in the implementation of blinding, suggesting that many studies did not implement blinding for participants, researchers, or outcome assessors, which increased the risks of performance bias and detection bias. The studies showed good performance in terms of data integrity, with a low proportion of high risk. Future studies should strengthen the application and reporting of blinding to further improve the quality of research (**Figure 3**).

Displays each domain for all 20 RCTs with colored “Low,” “Unclear,” and “High” risk icons. A legend explains colors, and each bar is labeled with the number of studies in that category.

### 3.3 Results of meta-analysis and meta-regression

#### 3.3.1 Total score of depressive symptoms

**Figure 4:**
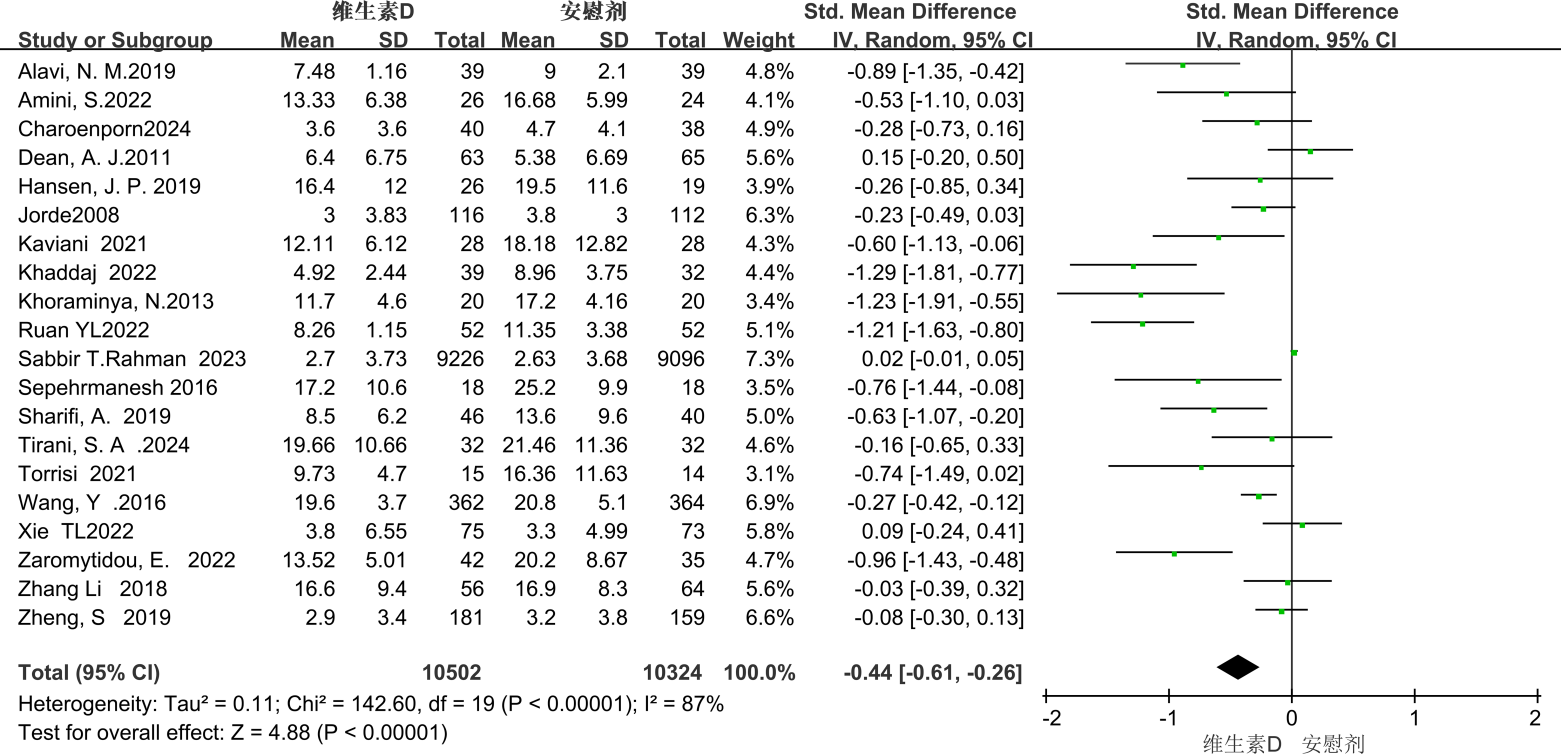
Forest plot of meta - analysis comparing the total scores of depressive symptoms between the two groups.

Depressive symptoms were rated in all 20 studies using the BDI, GDS, EPDS, PHQ, HDRS, DASS, and HAMD scales. Meta-analysis using a random-effects model showed that vitamin D supplementation had a statistically significant effect on depressive symptoms in patients with depression [SMD=-0.36, 95%CI (-0.52, -0.20), P<0.00001] (**Figure 4**).

#### 3.3.2 BDI score

**Figure 5:**
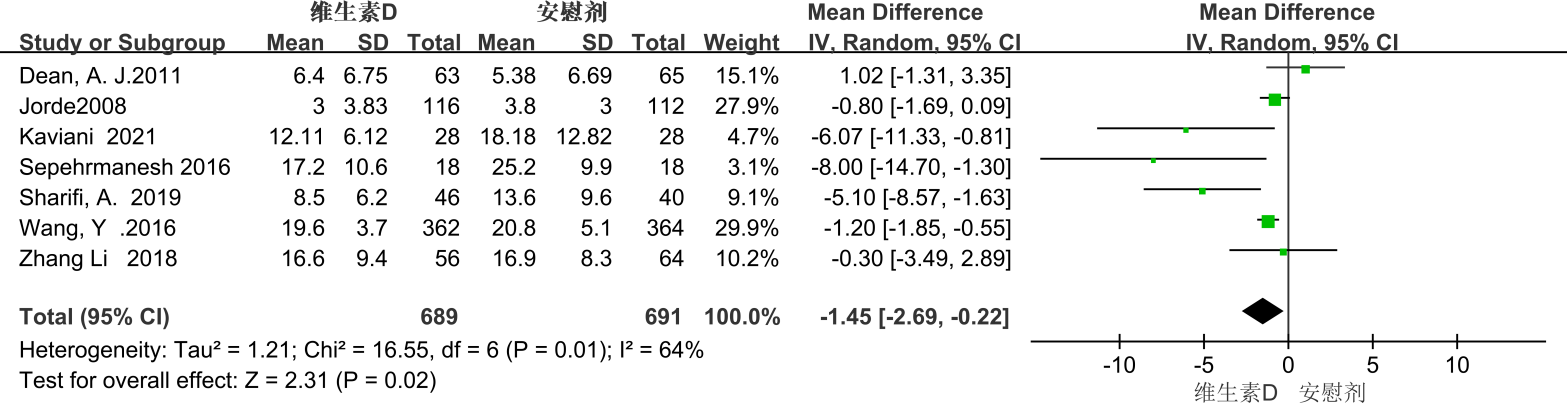
Forest plot of BDI scores.

Seven studies employed the BDI score as a metric for evaluation, revealing significant heterogeneity among the studies (P = 0.01, I² = 64%). Consequently, a random-effects model was utilized for the meta-analysis. The results showed that the BDI score of the experimental group was lower than that of the control group, indicating that vitamin D supplementation had a statistically significant effect on the alleviation of depressive symptoms in patients with depression [MD = -1.45, 95% CI (-2.69, -0.22), P = 0.02] (**Figure 5**). Due to the high heterogeneity, we performed subgroup analyses to identify sources of heterogeneity. Based on the characteristics of the included studies, we classified them by intervention duration. (See **Figure 5** for details).

##### 3.3.2.1 Duration of intervention

**Figure 6:**
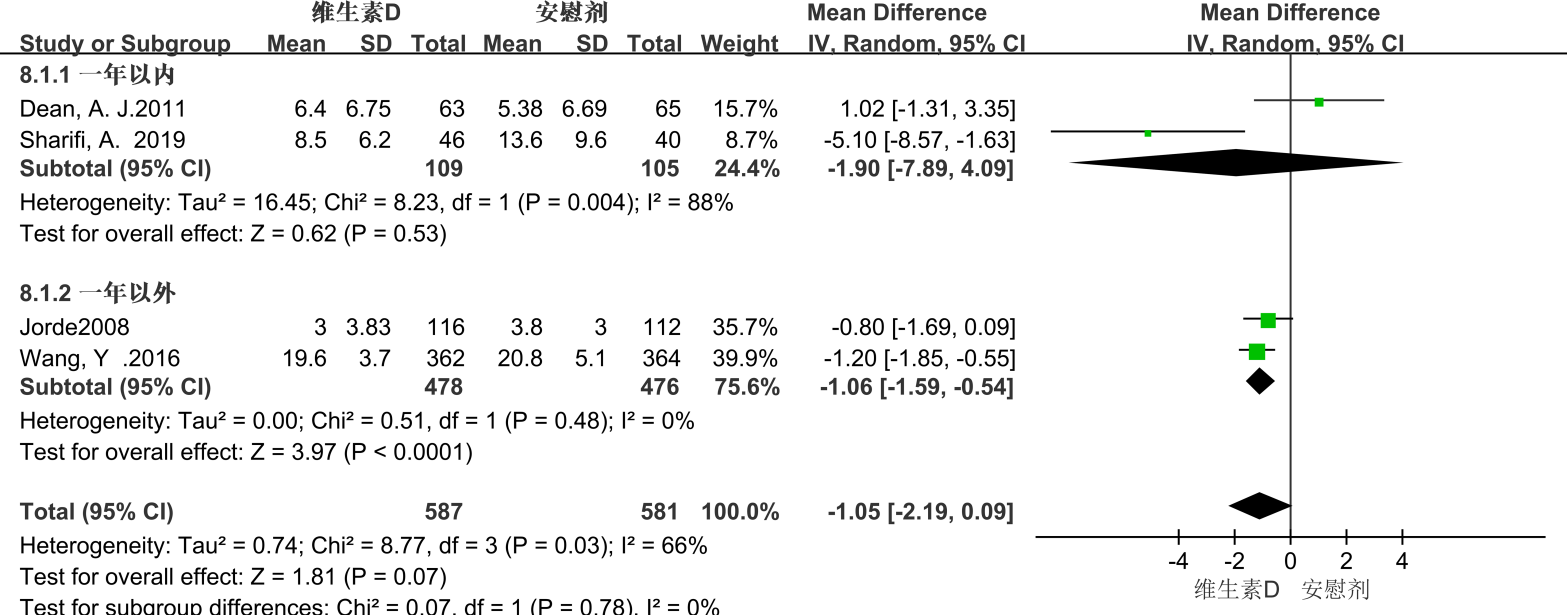
Subgroup analysis by intervention duration.

In the subgroup analysis conducted over a one-year timeframe, vitamin D supplementation showed a correlation with the reduction of depressive symptoms in patients experiencing depression for less than one year. However, this correlation did not reach statistical significance (MD = -1.90; 95% CI, -7.89 to 4.09; P = 0.53). In contrast, an analysis of interventions extending beyond one year revealed that vitamin D supplementation had a statistically significant relieving effect on depressive symptoms in patients with depression (MD = -1.06; 95% CI, -1.59 to -0.54; P < 0.0001). (See **Figure 6** for details).

#### 3.3.3 DASS score

**Figure 7:**
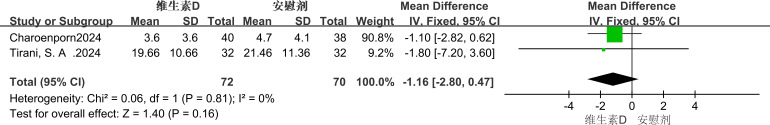
Forest plot of DASS scores.

The Depression, Anxiety and Stress Scale (DASS) provides a composite score integrating symptoms of depression, anxiety, and stress. Our meta-analysis revealed that vitamin D supplementation was associated with a numerically lower DASS total score compared to controls (MD = -1.16, 95% CI: -2.80 to -0.47), but this difference did not reach statistical significance (P=0.16). Importantly, this result specifically reflects the lack of a significant effect on the composite outcome; it does not preclude potential benefits of vitamin D on individual symptom domains (e.g., depression alone) that may require targeted evaluation. (See **Figure 7** for details).

#### 3.3.4 EPDS score

**Figure 8:**
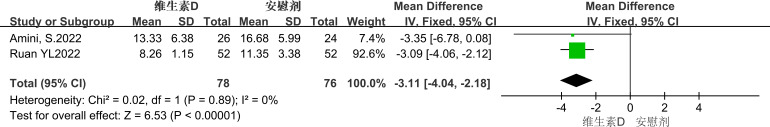
Forest plot of EPDS scores.

Two studies used the Edinburgh Postnatal Depression Scale (EPDS) score to evaluate the impact of vitamin D supplementation on depressive symptoms in pregnant women. The results showed that the EPDS score of the experimental group was lower than that of the placebo group (MD = -3.11, 95% CI: -6.78 to 0.08, P = 0.06), but this difference did not reach statistical significance. (See **Figure 8** for details).

#### 3.3.5 HAMD score

**Figure 9:**
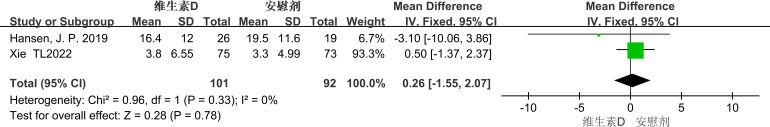
Forest plot of HAMD scores.

Two studies used the Hamilton Depression Rating Scale (HAMD) as the outcome measure, showing no significant heterogeneity (p = 0.33, I² = 0). A random-effect model analysis revealed no significant difference in alleviating depressive symptoms between the placebo group and the vitamin D supplementation group [MD = 0.26, 95% CI: -1.55 to 2.07, P = 0.78]. In these two studies, the duration of vitamin D supplementation was relatively short. In one study, the administration period was 12 weeks, and in the other, it was 2 months. (See **Figure 9** for details).

#### 3.3.6 PHQ-9 score

**Figure 10:**
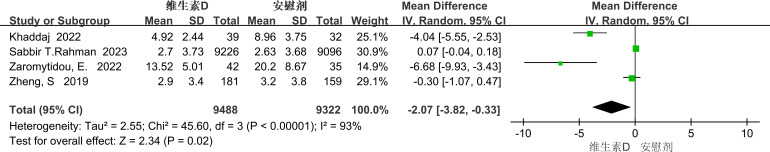
Forest plot of PHQ-9 scores.

The PHQ-9 score was used as an evaluative metric in four studies, which exhibited significant heterogeneity (p<0.00001, I²=93%). Due to this high heterogeneity, a random-effects model was employed to aggregate the effect sizes. The results revealed that vitamin D supplementation significantly alleviated depressive symptoms in individuals with depression [MD=-2.07, 95% CI: -3.82 to -0.33, P=0.02] (see **Figure 10**).

Given the substantial heterogeneity, we performed subgroup analyses to identify potential sources of this heterogeneity. Based on the characteristics of the included literature, we categorized the data by medical condition

Subgroup analysis

##### 3.3.6.1 medical illness

**Figure 11:**
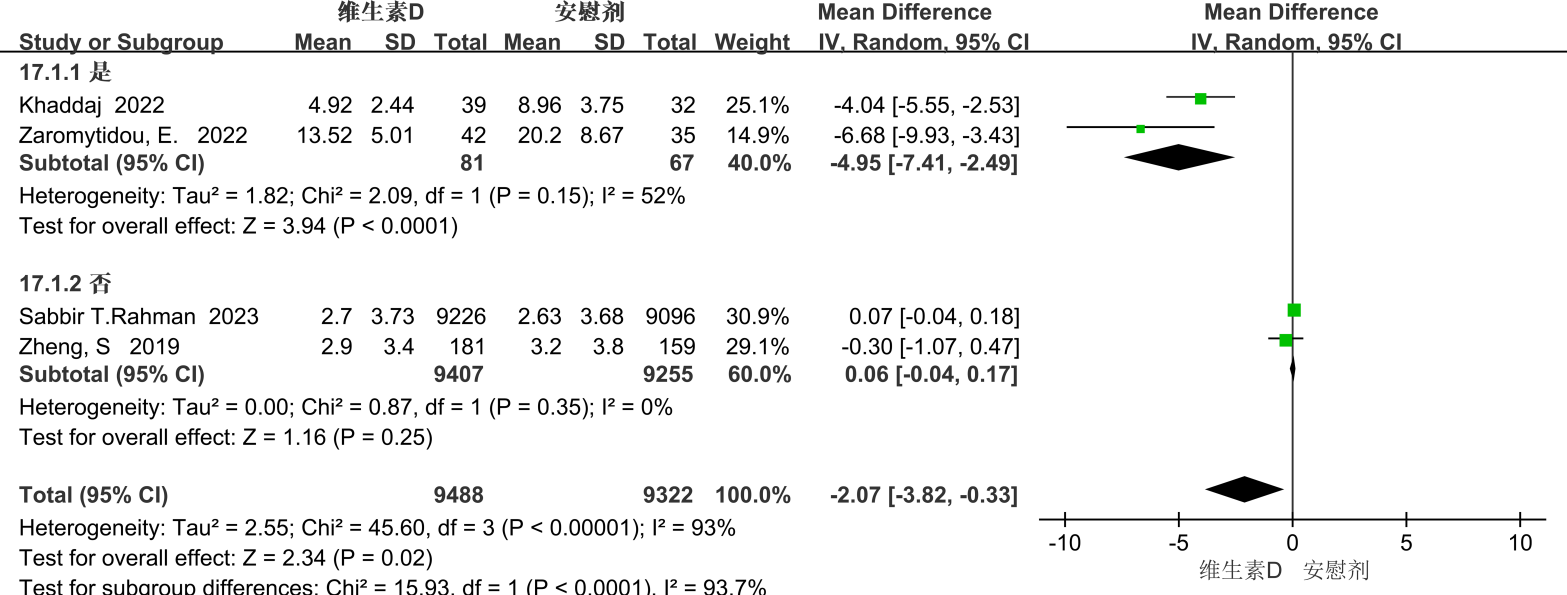
Forest plot by medical comorbidity.

In the subgroup analysis, the researchers grouped the patients according to whether they had comorbid medical (physical) conditions and explored the differences in the effects of vitamin D doses among different groups. The results showed that for patients with medical conditions, vitamin D significantly alleviated depressive symptoms (mean difference = -4.95; 95% confidence interval: -7.41 to -2.49; P = 0.0001). This indicates that vitamin D has a more significant effect on depressive patients with medical conditions. In contrast, in patients without medical conditions, the effect was not significant and there was no significant difference statistically (mean difference = -0.06; 95% confidence interval: -0.04 to 0.17; P = 0.25). (See **Figure 11**).

The overall results of the forest plot showed a significant reduction in depressive symptoms in the vitamin D supplementation group compared to the placebo group (standardized mean difference SMD = -0.36), with a 95% confidence interval of (-0.52 to - 0.20), which supported statistical significance (p <0.00001), but with a high heterogeneity I² of 83%. In subgroup analyses, the Beck Depression Inventory (BDI) showed significant improvement with a mean difference (MD) of - 1.45 (p < 0.05), although heterogeneity was high (I² = 64%). Further stratified by duration, long-term interventions (>1 year) were significantly effective (MD = -1.06, p < 0.0001), and short-term interventions (≤1 year) did not show significant differences; stratified by dose, low doses (<50,0000 international units) were significantly effective (MD = -1.04, p < 0.0001), and high doses (>50, 0000 IU) had no significant effect. The Patient Health Questionnaire-9 (PHQ-9) also showed significant improvement (MD = –2.07, P = 0.02), with an even stronger effect in patients with comorbid chronic diseases (MD = –4.95, P = 0.0001) and high consistency across studies (heterogeneity not reported). In contrast, no significant improvements were observed with the other scales (DASS, EPDS, HAMD). When stratified by population characteristics, vitamin D supplementation produced a significant reduction in depressive symptoms among patients with comorbid medical conditions (PHQ-9 subgroup: MD = –4.95). In contrast, the effect was smaller and did not reach statistical significance in healthy individuals (as reflected by DASS and EPDS scores), and there was no significant improvement in pregnant women (EPDS).

#### 3.3.7 Meta-regression for the different depression statuses

**Figure 12:**
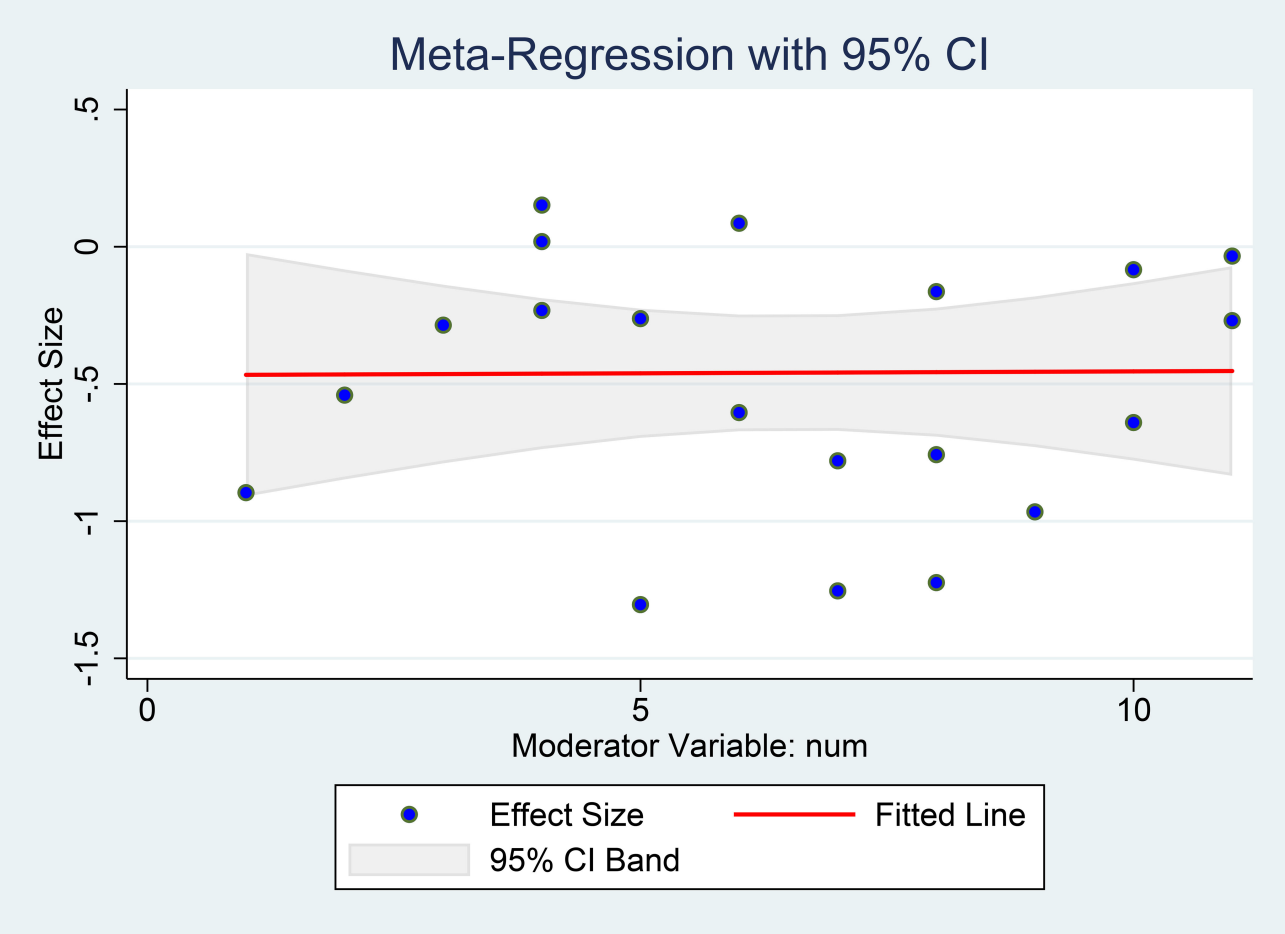
Meta-regression for the different depression statuses. X-axis shows each study’s mean baseline depression score (the moderator), Y-axis shows the study’s effect size (SMD). The red line is the fitted slope with its 95% CI.

We performed a meta-regression study to examine the potential influence of varying baseline depression statuses on the effects of vitamin D supplementation on depression scale outcomes. The results indicated no evidence that the influence of vitamin D supplementation on depression scores was contingent upon individuals’ baseline depression condition (coefficient=-0.468, SE=0.259, P=0.087, **Figure 12**).

## 4 Discussion

### 4.1 Summary of main findings

This meta-analysis of 20 RCTs demonstrated that vitamin D supplementation significantly alleviates depressive symptoms, with an overall standardized mean difference (SMD) of -0.36 (95% CI: -0.52 to -0.20, p < 0.00001), supporting its role as a viable adjunctive therapy for depression. Subgroup analyses revealed enhanced efficacy in long-term interventions (>1 year; MD = -4.95, p = 0.0001) and populations with medical comorbidities. Notably, patients with ulcerative colitis (UC) and dialysis-dependent individuals exhibited marked improvements in PHQ-9 scores (MD = -2.07, p = 0.02), underscoring the therapeutic potential in inflammatory or metabolic disorders. However, the observed –2.07-point reduction on the PHQ-9 did not reach the Minimal Clinically Important Difference (MCID) of 5 points, indicating the need for further trials to confirm a clinically meaningful benefit.

### 4.2 Mechanistic insights

Our findings are both consistent and divergent from the existing literature. Consistent with the study by Wang et al. (2016) as well as preclinical models, vitamin D exhibited anti-inflammatory (e.g., decreased tumor necrosis factor -α (TNF-α) levels) and neuroprotective effects (e.g., up-regulation of brain-derived neurotrophic factor (BDNF)/neuronal differentiation factor (NeuroD1)), confirming its role in attenuating neuroinflammation and enhancing synaptic plasticity (27). In addition, the efficacy of vitamin D in vascular depression is associated with improved cardiovascular status, which is consistent with the multi-target hypothesis.

Existing studies have found that the differences in the response to vitamin D between BDI/PHQ-9 and HAMD/EPDS may stem from the different emphases of these scales on the dimensions of depressive symptoms: BDI/PHQ-9 focuses on cognitive-affective symptoms (such as guilt, anhedonia) and physical fatigue, which is more consistent with the anti-inflammatory (such as TNF-α inhibition) and neuroprotective (such as BDNF upregulation) mechanisms of vitamin D. In contrast, HAMD/EPDS pays more attention to physical symptoms (such as insomnia) or unique emotional problems during the postpartum period (such as anxiety about the mother-infant relationship), and these symptoms may require longer-term intervention or multidimensional treatment. Consistent with previous meta-analyses, the efficacy of vitamin D is threshold-dependent (only effective for those with deficiency) and dose-sensitive (long-term supplementation with a low dose is more optimal). However, this study further reveals the impact of scale specificity on the results—BDI/PHQ-9 may be more suitable for biomarker-driven interventions, while the negative results of HAMD/EPDS may be related to the multifactorial etiology of postpartum depression (such as hormonal fluctuations) and insufficient dosage.

In BDI - scale - related studies, baseline levels and target - reaching thresholds have a significant impact on the efficacy. Take patients with ulcerative colitis (UC) as an example. When the vitamin D level is increased to 40.8 ng/mL through a single injection (exceeding the threshold of 30 ng/mL), the depressive state of patients has been significantly improved. In contrast, in the pulmonary tuberculosis (PTB) study, the vitamin D level was only increased to 27.1 ng/mL, so no effect was shown. This indicates that when the human body is in a state of severe vitamin D deficiency, the mechanism for improving depressive symptoms may be achieved through metabolic/antioxidant pathways. When the vitamin D level is close to a sufficient state, it mainly relies on the neuromodulation mechanism. In UC patients, the decrease in TNF - α is correlated with the improvement in BDI (Wang. Y, 2016). For dialysis patients, although long - term supplementation of vitamin D can significantly increase the vitamin D level in their bodies, it is only effective for vascular depression. This may be related to the improvement of cardiovascular complications (such as arteriosclerosis and calcium - phosphorus metabolism disorders), rather than directly from anti - inflammatory effects or nutritional support. It is worth noting that even if the vitamin D level in healthy people reaches the standard, depressive symptoms still do not improve, which highlights the importance of pathological needs. In terms of intervention programs, a single high - dose intramuscular injection (such as 300,000 IU in the UC study) can quickly bring the vitamin D level up to the standard, while long - term oral administration (such as 50,000 IU/week for more than 12 weeks in the dialysis study) requires a cumulative effect to play a role. In addition, combined calcium supplementation may enhance the regulation of the calcium - PTH axis, but this may also confound the independent effect of vitamin D. The current research design has certain limitations, such as insufficient sample size and lack of stratification of baseline levels. These factors may mask the efficacy of subgroups or overestimate short - term effects. In addition, depressive subtypes and genetic polymorphisms will further affect the efficacy of vitamin D, among which vascular depression is the most sensitive to vitamin D. Therefore, formulating a precise intervention strategy requires comprehensive consideration of multiple factors such as baseline levels, disease mechanisms, and individual differences.

In DASS - scale - related research, two studies have revealed the mechanisms of action of vitamin D from different perspectives. The PCS study found that vitamin D3 may improve fatigue by regulating neurotransmitters (promoting the expression of TPH2 and BDNF). The CFQ - 11 score decreased by 3.5 points, and its clinical significance remains to be verified in combination with the MCID threshold. However, this study failed to distinguish the differences in the bioavailability of D2 and D3 and lacks evidence of the penetration of the blood - brain barrier. The migraine study focuses on the gut - brain axis. The combination of probiotics can reduce the attack frequency by increasing the utilization rate of tryptophan, but has no effect on the duration of attacks, which may be due to selectivity in the intervention timing or target. Although the mechanisms of these two studies are different (neurotransmitter and microbiota - serotonin pathway), they both face contradictions in inflammatory markers. Vitamin D may play a non - classical anti - inflammatory role by regulating the NLRP3 inflammasome and other means. Moreover, there is gender bias (such as the regulation of estrogen on the CYP24A1 enzyme), so stratified analysis is required. It is recommended to break through the transformation bottleneck through dynamic monitoring of biomarkers (continuously measuring vitamin D levels and fecal microbiota) and gender - specific design, and promote the transition of vitamin D from a “broad - spectrum supplementation” to a “precision - targeted” strategy (27, 28).

Two studies explored the impact of vitamin D supplementation on postpartum depression (PPD), but both had limitations in terms of dosage and mechanism interpretation. The study by Amini, S. (2022) found that supplementing 50,000 IU of vitamin D3 every two weeks after childbirth could improve PPD symptoms, yet the serum vitamin D level still did not reach the sufficient threshold after the intervention. This implies that the dosage needs to be optimized (such as 50,000 IU per week or extending the cycle to ≥12 weeks), and a separate calcium supplementation group should be added to rule out interference. The exploration of the mechanism in this study only focused on inflammatory indicators, without involving neurotransmitters (such as serotonin, BDNF) and VDR gene polymorphisms, and the sample only covered people with vitamin D deficiency. Ruan YL. (2022) compared the effects of vitamin D supplementation before and after childbirth. However, in the prenatal study, due to unclear dosage, failure to control confounding factors such as social support and obstetric history, and relying solely on the Edinburgh Postnatal Depression Scale (EPDS) scores and breastfeeding rates, lacking mechanistic indicators, the credibility was low. In the postpartum study, due to the failure to unify the PPD assessment scale and the neglect of breastfeeding indicators, the results were difficult to compare. In both studies, the serum vitamin D level did not reach a sufficient level.

The combined treatment of vitamin D and fluoxetine has shown remarkable effects in improving depressive symptoms. However, only one small randomized controlled trial (Khoraminya et al., 2013; n = 42) has evaluated the efficacy of vitamin D plus fluoxetine, which showed superior symptom reduction compared with fluoxetine alone. Given the limited sample size and small study size, these findings are preliminary and future larger trials should be conducted to confirm any synergistic effects. A double-blind, randomized, placebo-controlled trial demonstrated that patients who took 1500 IU of vitamin D_3_ daily along with 20 mg of fluoxetine showed a significant improvement in depressive symptoms after the fourth week of treatment. Moreover, patients with lower baseline vitamin D levels had more severe depressive symptoms, indicating a correlation between vitamin D deficiency and the severity of depression. In addition, the combined supplementation of probiotics and vitamin D also has a positive impact on the depressive symptoms of migraine patients. A randomized, triple-blind, placebo-controlled trial found that patients who received 50,000 IU of vitamin D every two weeks and 4.5 × 10¹¹ colony-forming units (CFU) of probiotics daily showed a significant improvement in depressive symptoms after 12 weeks, but there was no significant impact on anxiety and stress. Overall, the supplementation of vitamin D and probiotics has the potential to improve depressive symptoms, especially when used in combination with other treatment methods (such as antidepressant medications). However, their impact on other mental health indicators still requires further research. Future studies should further explore the effects of vitamin D and probiotics, either alone or in combination, on the mental health of different populations to optimize treatment regimens.

Vitamin D is a recognized immunomodulator (30, 31), and its active form, calcitriol (1,25(OH)_2_D_3_), plays a significant role. It can inhibit the adaptive immune system and actively modulate the innate immune system, which in turn increases the phagocytic activity of immune cells while reducing the expression and release of inflammatory cytokines (32). Especially in the central nervous system, calcitriol can inhibit the activation of the NF-κB pathway and have a damaging effect on the expression of iNOS, pro-inflammatory TLR, NLRP3 inflammasome components, and pro-inflammatory cytokines, thereby preventing the activation of microglia and astrocytes and alleviating neuroinflammation (33–36). In addition, calcitriol can also impact the gut microbiota composition, promoting the physical barrier established by intestinal endothelial cells by strengthening intercellular connections and reducing intestinal permeability (37–39). Our benefits in the BDI and PHQ-9 subgroups are consistent with the anti-inflammatory and neurotrophic effects of vitamin D. Calcitriol inhibits the activity of NF-κB and NLRP3 inflammasomes, which may preferentially improve anhedonia and low energy (captured by BDI/PHQ-9) rather than the somatic/insomnia symptoms highlighted in HAMD/EPDS. This mechanistic link helps explain why we observed significant effects of BDI/PHQ-9 in the HAMD or EPDS subgroups but not in the EPDS subgroups.

Possible mechanisms between vitamin D and depression include the neurotrophic hypothesis. The widespread distribution of vitamin D receptors (VDR) and activating enzymes, along with 1,25-dihydroxyvitamin D_3_ (1,25(OH)_2_D_3_), regulates the expression of neurotrophic substances, affects neuron-related processes, and is associated with the onset of depression. The monoamine neurotransmission hypothesis posits that vitamin D deficiency affects the synthesis of monoamine neurotransmitters and neuronal development. It may lead to delayed differentiation of dopamine (DA) cells and behavioral defects through the loss of VDR expression. In terms of neuroimmunomodulation, 1,25(OH)_2_D_3_ inhibits the abnormal activation of the immune system. Vitamin D deficiency increases inflammatory markers in rats, and vitamin D supplementation has an antidepressant effect (40).

Vitamin D also maintains the health of brain function by regulating neural development (such as promoting neuronal differentiation and synaptic plasticity), balancing neurotransmitters (such as dopamine, serotonin, and the glutamate/γ-aminobutyric acid (GABA) system), and inhibiting neuroinflammation and oxidative stress. Its deficiency can trigger abnormal activities in the core regions of the default mode network (DMN), including the posterior cingulate gyrus and the angular gyrus, as well as functional disorders in the prefrontal-limbic system. This leads to excessive activation of self-referential thinking and a decline in executive control ability, thereby impairing cognitive functions such as working memory and sustained attention. There are significant gender differences. Due to factors such as the storage of vitamin D in subcutaneous fat, fluctuations in estrogen, and sun protection habits, women are more likely to be deficient in vitamin D. Low levels of vitamin D may exacerbate the cognitive impairment and emotional dysregulation in female patients with depression by affecting the fractional amplitude of low-frequency fluctuations (fALFF) in the DMN and the prefrontal lobe (41).

Vitamin D3 alleviates neuroinflammation and oxidative stress by inhibiting the excessive activation of hippocampal astrocytes induced by coal dust exposure (manifested as a downregulation of glial fibrillary acidic protein (GFAP) expression). Meanwhile, it significantly increases the distribution of brain-derived neurotrophic factor (BDNF) in the CA1/CA3 regions of the hippocampus and the expression of neuronal differentiation factor (NeuroD1), enhancing neural plasticity and the survival ability of neurons. Additionally, by upregulating the vitamin D receptor (VDR) signaling pathway, it further enhances its anti-inflammatory, antioxidant, and synaptic regulation effects. These improvements at the molecular level ultimately reverse hippocampus-dependent behavioral abnormalities (such as anxiety- and depression-like behaviors), indicating that vitamin D3 protects the structure and function of the hippocampus through a multi-target mechanism, providing important experimental evidence for the prevention and treatment of coal dust-related neuropsychiatric diseases (42).

Inflammation also plays an important role in depression. Inflammatory markers (C-reactive protein (CRP) or interleukin-6 (IL-6)) can predict future depression (43). Neuroinflammation is triggered by various factors, leading to the activation of microglia and astrocytes. The release of pro-inflammatory mediators affects neural plasticity and neuronal survival and disrupts the blood-brain barrier (44). The peripheral immune system is closely related to neuroinflammation. Some patients with depression have abnormal peripheral immune function, with an imbalance between T helper 17 cells (Th17) and regulatory T cells (Tregs). Moreover, the relationship between inflammation and depression varies among adolescents and genders. The gut microbiota interacts with the brain through the microbiota-gut-brain axis. Its imbalance can lead to intestinal inflammation, allowing bacterial products to enter the brain and trigger neuroinflammation, while improving the gut microbiota can alleviate depressive symptoms (45). Probiotics can regulate the gut microbiota and the immune system and improve depressive symptoms (46, 47). One study in this experiment confirms this view.

The gut microbiota and vitamin D influence each other through a two-way regulatory mechanism: The microbiota enhances the intestinal barrier function through probiotics (such as Lactobacillus and Bifidobacterium), promoting the absorption of vitamin D. Its metabolites, short-chain fatty acids (SCFAs), can activate the vitamin D receptor (VDR) and facilitate the conversion of the vitamin D precursor (25-OH-D) into its active form (1,25-(OH)_2_D_3_) (48, 49). At the same time, microbiota dysbiosis will exacerbate inflammation and deplete vitamin D, while SCFAs reduce pro-inflammatory factors (such as IL-6 and TNF-α) by inhibiting the NF-κB pathway, optimizing the metabolic environment. Vitamin D, by activating the VDR, promotes the secretion of antimicrobial peptides (such as defensins), inhibits pathogenic bacteria (such as Proteobacteria), supports the proliferation of beneficial bacteria, and reduces the entry of endotoxins (LPS) into the bloodstream by enhancing intestinal tight junction proteins (occludin and ZO-1), maintaining the homeostasis of the microbiota. The two work synergistically to regulate neurotransmitters (such as 5-HT and GABA), immune balance (Th17/Treg), and the function of the gut-brain axis, thereby affecting neurodegenerative diseases (such as the deposition of β-amyloid in Alzheimer’s disease), mental disorders (such as depression), and metabolic syndrome (such as insulin resistance).Clinical intervention recommendations include the combined supplementation of vitamin D (with a target serum 25(OH)D level > 30 ng/mL) and probiotics (Lactobacillus/Bifidobacterium), supplemented with a high-fiber diet to increase SCFAs, in order to improve the intestinal barrier, reduce inflammation (IL-6 and cortisol), and regulate metabolic functions (50).

In the anti-inflammatory treatment of depression, antidepressant drugs such as tricyclic antidepressants, fluoxetine, and ketamine have anti-inflammatory effects, but their specific mechanisms still need further study. Some studies have shown that the combination of vitamin D and fluoxetine is more effective than fluoxetine alone, and one study in this experiment also confirms this view. Among anti-inflammatory drugs, selective cyclooxygenase-2 (COX-2) inhibitors have shown therapeutic potential in preclinical studies; however, in clinical studies, the relationship between them and inflammatory mediators remains unclear. In addition, the efficacy and safety of non-COX-selective drugs need to be further verified through more clinical trials (51, 52). Moreover, physical activity, agmatine, probiotics, vitamin C, and vitamin D, etc., also have anti-inflammatory properties and may improve depressive symptoms. Physical activity exerts anti-inflammatory and antidepressant effects by regulating the NLRP3 inflammasome pathway (53). Low to moderate-intensity physical activity seems to have anti-inflammatory effects, but high-intensity physical activity is associated with inducing inflammation by increasing circulating pro-inflammatory cytokines (54, 55).

### 4.3 Limitations and future research directions

This study reveals the potential association between vitamin D supplementation and the improvement of depressive symptoms, but there are still significant limitations that require careful interpretation. The existing evidence shows a high degree of heterogeneity (I² = 83%), which may be due to explored confounding factors: ① At the biological level, the effects of vitamin D dosage forms, administration frequencies, and genetic polymorphisms (such as VDR gene variations) on metabolism have not been systematically investigated; ② At the environmental level, there is a lack of quantitative control over sunlight exposure, types of physical activities, and seasonal changes; ③ Methodological flaws are prominently manifested as the absence of baseline 25 (OH) D data, inadequate implementation of randomization and blinding, and limited sample representativeness (focused on specific populations such as pregnant women). These factors together limit the extrapolation of the results and may lead to publication bias that overestimates the actual effect.

Future research needs to establish a multi-dimensional innovation system: At the basic mechanism level, genetic testing (VDR/CYP24A1 polymorphisms), dynamic biomarkers (inflammatory factors, gut microbiota), and advanced neuroimaging techniques (7T MRI/fNIRS) should be integrated to establish a multi-omics efficacy prediction model; At the clinical practice level, it is recommended to conduct multicenter RCTs to verify phased dose adjustments (such as different regimens for prenatal/postpartum periods), cross-disease combination therapies (depression + migraine), and chronotherapeutic strategies (seasonal supplementation); At the public health level, a precise screening pathway based on the vitamin D metabolic pathway needs to be established to promote the transformation from the “broad-spectrum supplementation” model to the “mechanism-guided precision psychopharmacology” model (56, 57).

To achieve this transformation, three key breakthroughs are required: ① Develop an analysis platform for gene-environment interactions to analyze the impacts of skin pigmentation and regional cultural differences on treatment efficacy; ② Build a real-time monitoring system to quantify sunlight/physical activity exposure through wearable devices; ③ Innovate intervention designs and explore cutting-edge solutions such as the combined use of vitamin D and probiotics, and individualized dosing based on epigenetic clocks. These breakthroughs will help establish a precise intervention system featuring the trinity of “biomarker guidance - visualization of neural functions - dynamic dose regulation” (58–60).

These findings have many practical implications. Routine screening for vitamin D deficiency in patients with depression, especially those with chronic inflammatory or metabolic diseases, may identify those most likely to benefit from vitamin D supplementation. Our dose-response meta-regression analysis showed that higher cumulative doses over the long term resulted in better efficacy, and thus suggests that at least 50,000 IU per week for 6 to 12 months be administered to individuals with vitamin D deficiency. Furthermore, the failure to achieve the minimal clinically important difference (MCID) on the PHQ-9 questionnaire highlights that vitamin D alone may not be sufficient to treat moderate to severe depression, suggesting that combination therapy (e.g., with antidepressants or anti-inflammatory drugs) should be considered in future trials. Finally, precision psychiatry approaches, such as the integration of vitamin D receptor (VDR) polymorphisms, baseline cytokine profiles, and neuroimaging biomarkers, may improve patient selection and optimize dosing in the future.

## 5 Conclusion

This meta-analysis demonstrates that vitamin D supplementation significantly alleviates depressive symptoms (SMD = -0.36), particularly in subgroups with baseline deficiency (<20 ng/mL) and comorbid chronic inflammatory conditions. The therapeutic mechanism likely involves dual modulation of the neuro-immune axis (e.g., NLRP3 inflammasome suppression) and achievement of a threshold serum 25(OH)D level (>30 ng/mL).For clinical practice, we propose an individualized regimen targeting treatment-resistant depression: Dosage: 50,000 IU/week for ≥1 year (target serum 25(OH)D >40 ng/mL).High-priority populations: Patients with metabolic syndrome, seasonal affective disorder, or autoimmune diseases. Risk mitigation: Monitor hypercalcemia in renal insufficiency or CYP24A1 mutation carriers. Future research should: Elucidate molecular mechanisms by integrating VDR polymorphisms with single-cell sequencing (e.g., astrocyte-specific pathways).Develop precision dosing tools via medical big data-driven predictive modeling.

Address current limitations: Standardize 25(OH)D assays to reduce heterogeneity (I^2^ = 83%). Combine dynamic biomarker monitoring (e.g., inflammatory cytokines, calcium levels) with stratified trial designs.

While vitamin D emerges as a promising “metabolic-immune-neurological” modulator, its translation requires tailored strategies within precision psychiatry frameworks.

## Supplementary material

Supplementary Table 2 Supplementary table information included in the literature.
