# Supplementary material for: Meta-analysis of the effect of vitamin D on depression
Source: Front Psychiatry. 2025 Jul 31;16:1622796. doi: 10.3389/fpsyt.2025.1622796 (PMC12352333; doi:10.3389/fpsyt.2025.1622796)
Supplement: Supplementary file 2 [file DataSheet1.pdf]

Pubmed		Total
#1	"Vitamin D"[Mesh]	71249
#2	"Depression"[Mesh]	162207
#3	("Depression"[Mesh]) OR ((((((Depression[Title/Abstract]) OR (Depressive Symptoms[Title/Abstract])) OR (Depressive Symptom[Title/Abstract])) OR (Symptom, Depressive[Title/Abstract])) OR (Emotional Depression[Title/Abstract])) OR (Depression, Emotional[Title/Abstract]))	516673
#4	("Vitamin D"[Mesh]) AND (("Depression"[Mesh]) OR ((((((Depression[Title/Abstract]) OR (Depressive Symptoms[Title/Abstract])) OR (Depressive Symptom[Title/Abstract])) OR (Symptom, Depressive[Title/Abstract])) OR (Emotional Depression[Title/Abstract])) OR (Depression, Emotional[Title/Abstract])))	796

Cochrane Library		Total
#1	MeSH descriptor: [Vitamin D] explode all trees	7967
#2	(Depression):ti,ab,kw OR (Depressive Symptoms):ti,ab,kw OR (Symptom, Depressive):ti,ab,kw OR (Depressive Symptom):ti,ab,kw OR (Depression, Emotional):ti,ab,kw	112140
#3	(Emotional Depression):ti,ab,kw	8814
#4	#2 OR #3	112140
#5	#4 AND #1	208

Embase		Total
#1	'vitamin d':ab,ti	126881
#2	'depression'/exp	704401
#3	'central depression':ab,ti OR 'clinical depression':ab,ti OR 'depressive disease':ab,ti OR 'depressive disorder':ab,ti OR 'depressive episode':ab,ti OR 'depressive illness':ab,ti OR 'depressive personality disorder':ab,ti OR 'depressive state':ab,ti OR 'depressive symptom':ab,ti OR 'depressive syndrome':ab,ti OR depressivity:ab,ti OR 'mental depression':ab,ti OR 'parental depression':ab,ti OR depression:ab,ti	648279
#4	#2 OR #3	936083
#5	#1 AND #4	2830

中文数据库		Total
知网	（关键词：维生素 D(精确)）AND（关键词：抑郁(精确)）	1026
万方	检索表达式： 题名或关键词:(维生素 D) and 题名或关键词:(抑郁)	
维普	检索表达式： 题名或关键词=维生素 D and 题名或关键词=抑郁	
