# Supplementary material for: Meta-analysis of the effect of vitamin D on depression
Source: Front Psychiatry. 2025 Jul 31;16:1622796. doi: 10.3389/fpsyt.2025.1622796 (PMC12352333; doi:10.3389/fpsyt.2025.1622796)
Supplement: Supplementary file 3 [file DataSheet2.pdf]

Study, Year	No. of Participants in the Meta – Analysis		Duartion of Intervention	Intervention Measures	Scale	Scale Score			
						Scale Baseline		Baseline VD Content	
	Total Staff	Depression Type				Vitamin D Group<br>Mean(SD)	Placebo Group<br>Mean(SD)	Vitamin D Group<br>Mean(SD)	Placebo Group<br>Mean(SD)
Khaddaj, 2022	71	Mild to severe depression	3 Months	50,000 IU per week (in daily diet)	PHQ - 9	12.53(5.33)	12.4(4.66)	16.92(7.23)	16.44(5.91)
Sabbir T.Rahman et al., 2023	18322	With depressive tendency	5 Years	60,000 IU per month (once a month)	PHQ - 9	2.66 (3.68)	2.66 (3.78)		
Zaromytidou, E. et al., 2022	77	Depressive tendency with comorbid somatic illness	12 Months	Once a week, take the oral solution form of VD (25,000 IU) or receive no intervention at all	PHQ - 9	43.85(9.40)	44.17(5.94)	19.98 (6.73)	19.85 (5.72)
Zheng, S et al., 2019	340	Mild to moderate depression with comorbid somatic illness	24 Months	Take 50,000 IU (1.25 mg) of vitamin D orally every month	PHQ - 9	3.4(4.1)	3.0(4.0)	43.7(11.8)	43.8(12.7)
Xie TL etal.,	148	Mild to	2 Months	All received	HAMD	30. 7(8.07)	29. 50(9. 62)	38.24(13.63)	41.05(14.28)

2022		moderate depression		conventional antidepressant treatment, and the intervention group was additionally supplemented with 1600 IU of vitamin D daily					
Khoraminya, N. et al., 2013	40	Major depressive disorder	8 Weeks	Take 1.5 tablets (1500 IU) of vitamin D and one tablet (20mg) of fluoxetine every day	HAMD 、HDRS	29.4(5.23)	30.2(5.83)	23.5(4.03)	23.0(4.42)
Hansen, J. P. et al., 2019	45	Mild to severe depression	Administer the drug for 12 weeks, and follow up for another 12 weeks	70 micrograms of vitamin D3 (2800 international units) capsules per day	HAMD -17、MDI	43.2 (24.6)	44.3 (24.1)	18 (6)	18 (6)
Dean, A. J. et al., 2011	128	With depressive tendency	6 Weeks	One capsule of 5000 IU every day	BDI	7.24（6.67）	5.72（6.69）	76.2 (20.64）	77.2 (20.96）
Jorde et al., 2008	228	With depressive	12 Weeks	Two capsules per week (one	BDI	4.5(4)	4(4.08)	55.2(13.37)	52.4(13.48)

		tendency		capsule containing 20,000 IU) or two placebo capsules					
Sepehrmanesh et al., 2016	36	Major depressive disorder	8 Weeks	50,000 IU vitamin D capsules per week	BDI	25.2（9.2）	28.5（10.8）	13.6（7.9）	9.2（6.0）
Kaviani et al., 2021	56	Mild to moderate depression	8 Weeks	Once a week, 50,000 IU of vitamin D3 or a placebo	BDI - II	23.86 (5.49)	21.79 (5.74)	87.1 (28.55)	73.64 (31.94)
Sharifi, A. et al., 2019	86	Mild to moderate depression with comorbid somatic illness	/	1 mL of 300,000 VD3 or 1 mL of normal saline (placebo) administered by intramuscular injection	BDI - II	12.82(5.59)	14.1(7.67)		
Wang, Y. et al., 2016	726	Major depressive disorder (MDD) with comorbid somatic illness	52 Weeks	Take 50,000 IU of vitamin D3 orally every week	BDI - II	22.7(4.3)	21.9(5.4)	21.9(4.1)	23.2(5.8)
Zhang Li et al., 2018	120	Major depressive	/	Take 100,000 IU orally every	BDI - II	23.5(4.03)	23.0(4.42)	24.6 (13.1)	23.3 (10.5)

		disorder (MDD) with comorbid somatic illness		week					
Charoenporn et al., 2024	78	Mild depression	8 Weeks	Take 60,000 IU of VD2 orally every week	DASS	5.6(3.7)	5.0(4.1)	18.9(5.4)	17.3(5.0)
Tirani, S. A. et al., 2024	64	Moderate to severe depression with comorbid somatic illness	12 Weeks	Take one pill containing 50,000 IU of vitamin D every two weeks	DASS	23.28(4.75)	24.35(11.11)		
Amini, S. et al., 2022	50	Postpartum depression	8 Weeks	50,000 IUVD per week + daily placebo Ca tablets	EPDS 、PPD	17.50(3.97)	16.43(3.07)	39.83(31.94	36.74(28.40,
Ruan YL et al., 2022	104	Moderate to severe depression with comorbid somatic illness	6 Months	The observation group was given vitamin D drops, administered once a day, with one capsule (400 IU) taken orally each time	EPDS	8.26(1.15)	11.35(3.38)	36.38(3.22)	36.24(3.16)
Alavi, N. M. et al., 2019	78	Moderate to severe depression	8 Weeks	50,000 units of VD per week	GDS - 15	9.25（2.4）	8.9(2.3)	22.57(6.2)	21.2（5.8）

Torrisi et al., 2021	29	Moderate to severe depression with comorbid somatic illness	12 Weeks	Intervention: Take one probiotic capsule daily + take 2000 IU of vitamin D orally every two weeks; Control: Take one placebo capsule containing starch and maltodextrin daily, and take one placebo pearl containing corn oil every two weeks.	MADRS	22.13(6.68)	21.8(10.43)	19.62(7.92)	14.05(4.9)
